# Commentary: Introduction to the Frontiers Research Topic: Optimization of Exercise Countermeasures for Human Space Flight–Lessons From Terrestrial Physiology and Operational Considerations

**DOI:** 10.3389/fphys.2019.00915

**Published:** 2019-07-19

**Authors:** Joseph J. Bevelacqua, James Welsh, S. M. J. Mortazavi

**Affiliations:** ^1^Bevelacqua Resources, Richland, GA, United States; ^2^Department of Radiation Oncology, Stritch School of Medicine, Loyola University, Chicago, IL, United States; ^3^Medical Physics Department, Shiraz University of Medical Sciences, Shiraz, Iran; ^4^Diagnostic Imaging, Fox Chase Cancer Center, Philadelphia, PA, United States

**Keywords:** exercise, space, astronauts, space radiation, oxidative stress, reactive oxygen species

This Commentary addresses the paper “Introduction to the Frontiers Research Topic: Optimization of Exercise Countermeasures for Human Space Flight–Lessons from Terrestrial Physiology and Operational Considerations” recently published by Scott et al. (2019). The authors of this well-structured paper have addressed the efficacy of exercise countermeasure (CM) and the importance of its optimization for all individuals. The authors considered the management of microgravity and adaptation to this well-known environmental stressor in space. Despite numerous strengths, the paper authored by Scott et al. has at least one shortcoming that comes from ignoring the key point that physical exercise has the potential to increase free radical production and lead to oxidative stress (Cooper et al., 2002). Given this consideration, as shown in Figure 1, while space flight can also trigger oxidative stress (Tian et al., 2017), physical exercise can significantly amplify the level of oxidative stress. Within a microgravity environment, it is possible to “overdo” the oxidative stressors and quickly transition from a beneficial range into a harmful range (especially when combination stressors coincide).

**Figure 1:**
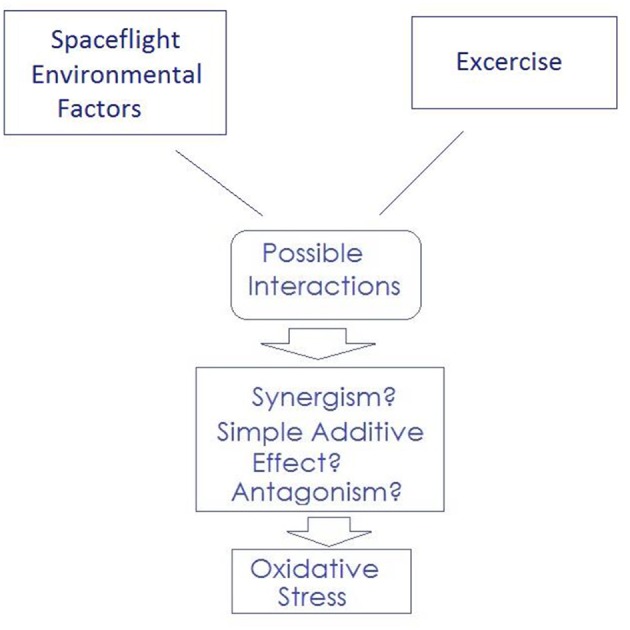
Both physical exercise and Spaceflight Environmental Factors can trigger oxidative stress. This combination can significantly amplify the level of oxidative stress.

Moreover, it has been shown that low-dose rate radiation exposure (e.g., ~100 mSv for half a year on the International Space Station, dose rate ~0.6 mSv/d) can lead to oxidative stress “*Results from astronauts participating in 4- to 6-month missions reveal increased carotid intima-media thickness (Arbeille et al.*, *2016**) and vascular stiffness (Hughson et al.*, *2016**) that are suggested to be related to increased oxidative stress, inflammation, and insulin resistance*” (Garrett-Bakelman et al., 2019). Moreover, Pavlakou et al. (2018) state “*Oxidative Stress in the environment of weightlessness and space irradiation has been shown in several tissue types like ocular tissue (Mao et al.*, *2013**), neural stem cells (Tseng et al.*, *2014**), as well as brain cortex and hippocampus (Mao et al.*, *2016**)*, *skin (Mao et al.*, *2014**), and intestine (Datta et al.*, *2012**). During the ESA-SPHINX (European Space Agency's-SPaceflight of Huvec: an Integrated eXperiment) experiment, the induction of oxidative stress response was shown after studying the impact of space environment exposure on 12 cell-kits of human umbilical vein endothelial cells (HUVECS)*.” However, in any space mission, there are many variables besides radiation and microgravity that can influence the experimental outcome. In addition, spacecraft environmental conditions (e.g., atmospheric pressure and composition, vibration, ambient lighting, and cabin non-ionizing radiation) could potentially influence the results. It may be possible to isolate the effects of a single variable (e.g., simulating gravity with a centrifuge), but the collective effects are more difficult to determine. Attempting to isolate each individual factor's particular impact is a possible, but difficult approach. Using that methodology, each of the effects of the numerous variables might then be estimated. In addition, a key question is when a specific level of exercise does not fit all astronauts, wouldn't it be logical that the same situation holds true for other space factors which cause oxidative stress?

Exercise in space is complicated because the normal body processes are perturbed by microgravity and the lack of a preferred direction provided by the Earth's gravitational field. Although a muscle can be exercised, the physiological processes associated with exercise are not directly comparable to exercise on Earth. In addition, the ambient environment of a space craft introduces additional factors (e.g., oxygen content, lighting, restricted volume, and electromagnetic background) that introduce additional perturbations.

Although exercise, in particular at high durations and intensities can become potentially harmful in space environment through increasing ROS/RNS production and negatively affecting oxidative stress balance, more caution should be considered in evaluation of low or moderate levels of exercise. While substantial evidence shows that prolonged or short-duration high intensity exercise can lead to increased radical production in active skeletal muscles resulting in the formation of oxidized lipids and proteins in the working muscles (Powers et al., 2016), moderate to low intensity exercise training is possibly beneficial for decreasing elevated levels of oxidative stress induced independently or combined by inactivity and hypoxia (Debevec et al., 2017). Regarding the role of the pattern and duration of exercise, some evidence also shows that high-intensity discontinuous exercise does not cause higher levels of exercise-induced oxidative stress compared to that of continuous moderate-intensity training (Vezzoli et al., 2014). It has also been reported that while very prolonged ultra-endurance exercise can lead to increased levels of ROS production, the dose-response curve always shows a linear relationship with the duration of exercise (Vezzoli et al., 2016).

Other issues that should be fully addressed are the possible interactions of physical exercise with other major stressors in space such as radiation (i.e., induction of simple additive or synergistic effects as a response to combined exposure to exercise and radiation). The aforementioned factors should be considered in assessing the conclusions of Scott et al. (2019).

## Author Contributions

All authors listed have made a substantial, direct and intellectual contribution to the work, and approved it for publication.

### Conflict of Interest Statement

JB was employed by company Bevelacqua Resources. The remaining authors declare that the research was conducted in the absence of any commercial or financial relationships that could be construed as a potential conflict of interest.
